# Draft Genome Sequence of the Bacterium *Gordonia jacobaea*, a New Member of the *Gordonia* Genus

**DOI:** 10.1128/genomeA.00995-15

**Published:** 2015-09-03

**Authors:** G. Jiménez-Galisteo, T. G. Villa, T. Vinuesa, M. Viñas, A. Domínguez, E. Muñoz

**Affiliations:** a Laboratory of Molecular Microbiology, Department of Pathology and Experimental Therapeutics, Medical School, University of Barcelona, Barcelona, Spain; b Department of Microbiology, University of Santiago de Compostela, Santiago de Compostela, Spain; c Department of Microbiology & Genetics, University of Salamanca, Salamanca, Spain; d Department of Cell Biology and Pathology, University of Salamanca, Salamanca, Spain; e CESPU-INNFACTS, Gandra, Portugal

## Abstract

*Gordonia jacobaea* was isolated and characterized in the Department of Microbiology, University of Santiago de Compostela, in 2000. Here we present the draft genome sequence of this species, which will improve our understanding of the diversity and the relation of the cell wall proteins of *G. jacobaea* with other mycolata.

## GENOME ANNOUNCEMENT

The actinomycete *Gordonia jacobaea* (1, 2) is a Gram-positive, non-spore forming, catalase-positive, and orange pigment-producing rod. Interest in the genus *Gordonia* resides in its ability to synthesize pigments and other substances of industrial interest and to degrade pollutants.

The genus *Gordonia* also has clinical interest since it includes almost 20 species, some of which act as opportunistic pathogens, such as *Gordonia terrae*, *G. aichiensis*, *G. bronchialis*, and *G. rubropertincta* (3). In addition, from a phylogenetic point of view the genus *Gordonia* is closely related to the actinomycetes CNM (*Corynebacterium*, *Nocardia*, and *Mycobacterium*), which synthesize large amounts of mycolic acids in the cell wall, and some of which are responsible for important human diseases such as leprosy and tuberculosis (4).

The extremely hydrophobic envelope of mycolata is a permeable barrier which makes the bacteria highly resistant to the action of antibiotics and other chemotherapeutic agents (5). In the last decades several investigators have pointed out the existence of proteins playing a similar role to that of the porins of Gram-negative bacteria, facilitating the diffusion of hydrophilic solutes through the bacterial envelopes and the entry of antibiotics (6, 7). The first description of these porins occurred in the 90s, when a porin of *Mycobacterium chelonae* was characterized (8); since then, other porins in genera like *Mycobacterium* (9, 10), *Tsukamurella* (11), or *Nocardia* (12) have been reported.

In our laboratories, we have isolated a pore-forming protein. To better understand how it works and seek relationships with other pore-forming proteins reported in other members of this bacterial group, we have sequenced the complete genome of *Gordonia jacobaea*.

Specifically, a 100-mL bacterial culture was grown in aerobic conditions at 30°C to the stationary phase in triptic soy broth. High-quality genomic DNA was extracted using the phenol-chloroform-isoamyl alcohol protocol.

To generate the shotgun sequencing library, genomic DNA (500 ng) was fragmented using compressed nitrogen gas according to the manufacturer’s standard protocol (GS FLX Titanium rapid library preparation kit, Roche Diagnostics). Both ends of the DNA fragments were blunt ended and ligated to DNA adaptors. Small fragments less than 350 bp were removed by using AMPure beads (Beckman Coulter). After small fragment removal, the library was assessed using the Agilent 2100 Bioanalyzer (Agilent Technologies). Finally, the DNA library was diluted to a working stock of 1 × 107 molecules/µL in Tris-EDTA (TE) buffer. The 454 pyrosequencing was performed with the GS Titanium sequencing kit XLR 70 (Roche Diagnostics) on a 454 GS-FLX sequencer (454 Life Sciences) following the manufacturer’s standard protocol. The 454 shotgun reads were assembled using GS De Novo assembler.

The draft genome sequence consists of 25 contigs with a mean GC content of 65.3% and a total length of 4,920,416 bases. Gene annotation was performed manually and we identified a total of 4,451 protein-coding, 47 tRNAs, 4 rRNAs, 5S, 16S, 23S, and 1 noncoding RNA (ncRNA).

### Nucleotide sequence accession number. 

The draft genome sequence of *G. jacobaea* strain MV1 has been deposited in GenBank under the accession number LDTZ00000000. The version described in this paper is the first version.
